# Caste-based social inequalities and childhood anemia in India: results from the National Family Health Survey (NFHS) 2005–2006

**DOI:** 10.1186/s12889-015-1881-4

**Published:** 2015-06-05

**Authors:** Priya Vart, Ajay Jaglan, Kashif Shafique

**Affiliations:** Department of Health Sciences, Community and Occupational Medicine, University Medical Center Groningen, University of Groningen, Groningen, The Netherlands; Department of Paediatrics, Medical College Guwahati, Guwahati, India; School of Public Health, Dow University of Health Sciences, Karachi, Pakistan; Institute of Health & Wellbeing, Public Health, University of Glasgow, 1-Lilybank Gardens, Glasgow, UK

**Keywords:** Caste, Anemia, Education, Wealth, Children

## Abstract

**Background:**

Caste is one of the traditional measures of social segregation in India and differs from other indicators as it is both, endogamous and hereditary. Evidence suggests that belonging to lower castes exposes one to social inequalities and affects health adversely. We examined the association of caste with childhood anemia in India and explored the effect modifying role of adult education and household wealth.

**Methods:**

A cross-sectional analysis of National Family Health Survey (NFHS) data of 43,484 children aged 6–59 months was performed. Poisson regression analysis was conducted to study the association between caste and childhood anemia accounting for various maternal, child, and household related variables. Caste was categorized as “other caste” (least disadvantageous), “other backward caste”, “scheduled tribe” and “scheduled caste” (most disadvantageous). Anemia was defined as mild (hemoglobin level 7-11 g/dL), moderate (hemoglobin level 5-7 g/dL) and severe (hemoglobin level <5 g/dL).

**Results:**

We found that children in scheduled caste had higher risk of having anemia [mild anemia: RR = 1.10, 95 % CI = 1.05-1.15; moderate anemia: RR = 1.19, 95 % CI = 1.14–1.24; severe anemia: RR = 1.87, 95 % CI = 1.51 – 2.31] after accounting for child, maternal and household covariates including adult education and household wealth. The interaction of caste with adult education and household wealth was not statistically significant for any level of anemia. Sensitivity analyses for children born to mothers of age ≥ 18 years at first child birth and body mass index (BMI) ≥ 18.5 kg/m^2^, resulted in similar findings.

**Conclusion:**

Caste is an independent determinant of childhood anemia in India. The level of adult education and household wealth did not modify the association between caste and childhood anemia. The findings may be used for countering childhood anemia and it may be beneficial to target future public health actions towards disadvantageous castes in India.

## Background

The proportion of children under 5 years of age having anemia in India is among the highest in the world. The prevalence of childhood anemia has been reported as high as 70 % in India [1]. Anemia is strongly associated with child morbidity and mortality [2]. Existing research on childhood anemia has mainly focused on factors like maternal age, education, height, child breastfeeding and place of residence [3]. Caste is one of the traditional measure of social segregation in India and differs from other indicators in that it is both endogamous and hereditary [4, 5]. Caste based social segregation has been linked to socioeconomic disadvantage and many adverse health outcomes [6, 7].

A large proportion of people of disadvantageous castes belong to low socioeconomic groups in India. Therefore, these people are generally exposed to poor living conditions, have poor diet and limited access to health care. Consequently, children born in disadvantageous castes are more likely to face infections (*e.g.* malaria), have iron deficient diet and limited availability of iron supplements which ultimately can lead to anaemia. In addition to their low socioeconomic circumstances, people in disadvantageous castes experience other adverse circumstances such as caste based discrimination. Caste based discrimination is associated with poor mental health which in turn has been shown to be associated with iron deficiency in infants [8]. Thereby, caste might be associated with anaemia independent of socioeconomic factors like education and wealth. Moreover, previous studies have also speculated about effect modification between caste and socioeconomic factors for their association with child mortality in India [9]. Unfortunately, the association between caste and childhood anemia in relation to adult education and household wealth is not well understood in India. Although a few studies has highlighted higher prevalence of childhood anemia in disadvantageous castes in India [10–14], these studies neither explored association between caste and childhood anemia independent of adult education and household wealth nor did they investigate effect modifying role of adult education and household wealth in this association.. Furthermore, previous studies were conducted on small non-representatives samples of regional populations, using non-probability-based sampling techniques which limited the external validities of those studies.

Therefore, we examined the association of caste based social segregation and childhood anemia while accounting for adult education and household wealth in a nationally representative sample of children under the age of 5 years in India. We further explored any effect modifying role of adult education and household wealth in the association between caste and childhood anemia.

## Methods

The data for this study was drawn from the National Family Health Survey – 2005–2006 (NFHS) held in India (data released in 2008). The NFHS was conducted to establish a representative data of demographic and health indicators of national and state population of India. In particular, child and maternal health outcomes were targeted. The NFHS is similar to Demographic Health Surveys (DHS) which are operational in a large number of developing countries. Details of the survey can be found at: http://www.measuredhs.com/.

In brief, this survey included children born to the mothers aged 15–49 years since January 2000/2001 (in some states data collection started in January 2000 and while in others in January 2001). A total of 124, 385 women, from 131, 596 eligible women (response rate 94.5 %) participated in the survey. Women were included from 116, 652 randomly selected households. Since January 2000/2001, 51, 555 live births (age 0 – 59 months) were reported. The protocol of NFHS was assessed and approved by ethics committee at the International Institute for Population Sciences, Mumbai in association with Ministry of Health, India.

### Study population and design

For the present study we included singleton children aged 6 – 59 months born in 29 states in India. Since hemoglobin levels were measured only for children aged ≥ 6 months, we restricted our sample to children aged 6 – 59 months (n = 43, 484). We excluded the children with missing information on anemia levels (n = 6,277), caste (n = 1,973), adult education (n = 154), and other children/maternal/household covariates including birth order, mother’s smoking status, mother’s occupation and household wealth (n = 1,937), leaving a sample of 33,488 children for final analysis. In baseline characteristics, no notable differences were observed between original and final study sample (data not shown but available from authors).

### Caste, adult education and household wealth

Information on caste was based on the type of caste self-reported by head of the household. Types of the castes were scheduled caste, scheduled tribes, other backward class and other caste [15].

Information on adult education was obtained from education levels of both parents. Adult education, thereby, refers to two variables *i.e.* maternal education and paternal education. Adult education was recorded according to the levels of education mentioned in Indian educational system *i.e.* no education (0 years), primary education (1–5 years), secondary education (6–10 years) and higher education (more than 10 years).

Household wealth was defined in terms of household assets and material possessions. Asset ownership is a reliable measure of wealth and living standards in India [16]. We use the wealth score provided in the NFHS 2005–2006 dataset. In this dataset, wealth score was obtained based on a combination of household characteristics including consumer durables, characteristics of the residential place and land ownership. Household characteristics were weighted according to a factor analysis procedure. Households were allocated a final household wealth score calculated from the summation of weights for each item into a linear index. Linear index was then converted into quintiles for the analysis.

### Anemia

Anemia was measured from hemoglobin levels of the children aged 6 – 59 months Hemoglobin levels of 7-11 g/dL, 5–7 g/dL and <5 g/dL were categorized as mild, moderate and severe anemia, respectively [17].

### Other covariates

Child covariates included were age, gender, birth order, religion and place of residence. Birth order of the child was categorized into three levels of birth order *i.e.* less than 3, 3 to 6 and more than 6. Religion of the child was determined based on the religion of the head of the household (Hindu, Muslim, Christian, other). Place of residence was classified into rural and urban. Maternal covariates included were age at birth of first child, body mass index (BMI), marital status, smoking and occupation. The height and weight of women were measured using a solar-powered electronic (SECA) scale with a digital screen. Body mass index was calculated using the standard formula-Weight (kg)/Height^2^ (m^2^). Marital status of the mother was categorized as married and unmarried (including never married, divorced, widowed or separated). Smoking status was defined as nonsmokers and smokers (smoking cigarettes/bidis and/or pipe/cigar). Maternal occupation was classified into non-worker, manual worker or non-manual worker.

### Statistical analysis

All analyses were conducted using STATA version 11 (StataCorp, College Station, TX, USA). A *p*-value of <0.05 was considered to indicate statistical significance. We used NFHS recommended specific weights that accounted for the sample weights and the multistage cluster survey sampling design. Baseline characteristics for continuous data was reported as mean (standard error). Categorical data are presented as weighted percentages with 95 % CI. Means are compared between groups using ANOVA and proportions were compared using Pearson’s chi^2^ test.

Since anemia was not a rare outcome in our study, we performed modified Poisson regression approach with robust error variance. This approach models the binary outcome and provide risk ratios with 95 % confidence internal [18]. We did separate analyses for mild, moderate and severe levels of anemia. First we estimated the independent association of individual anemia levels with caste (model 0). Subsequently, adjustment was made for potential confounders including child (age, gender, birth order and religion) and maternal demographic factors (age at birth of first child, BMI, marital status, occupation, smoking status and place of residence) (Model 1). In order to explore whether the association between anemia and caste existed after additionally adjusting for adult education (model 2) and household wealth (model 3) individually, and together (model 4), we re-estimated the models. Effect modification between caste and adult education and then between caste and household wealth was investigated from interaction terms.

We performed sensitivity analyses to test the robustness of our findings. First, understanding the significant association between maternal age at birth of first child and child nutritional status, we re-estimated the association between caste and anemia levels only for children who were born to mothers aged ≥ 18 years at the birth of their first child. Second, to account for disadvantage of having anemia in children born to mothers with low BMI, we repeated the analysis for children having a maternal BMI ≥ 18.5 kg/m^2^.

## Results

### Baseline characteristics

The baseline characteristics of the study population according to caste are presented in the Table 1. Almost one third of the study population (30.4 %) belonged to other caste (*i.e.* higher caste) and one fifth (19.3 %) to the scheduled casted (lowest caste). There was no difference in the mean age of the child among different castes (p = 0.107). In the scheduled tribe, prevalence of overall anemia was highest (77 %) and maternal BMI was lowest (19.3 kg/m^2^). Maternal smoking was similar in scheduled tribe and scheduled caste (2.5 % each). Most of the children in scheduled tribe were born in rural areas (91 %).

**Table 1:** Baseline characteristics of study population according to caste^a^

	Caste				
Child characteristics	Other caste	Other backward class	Scheduled tribe	Scheduled caste	*p*-value
(N = 33,488)	(n = 10,185)	(n = 11,819)	(n = 5,015)	(n = 6,469)	
**Age** (months)	32.7 (0.19)	32.8 (0.14)	32.7 (0.26)	32.5 (0.19)	0.107
**Male**	53 (52–55)	54 (53–55)	52 (50–54)	52 (50–53)	0.003
**Birth order**					<0.001
<3	80 (79–82)	73 (71–74)	64 (62–67)	71 (69–73)	
3 to 6	15 (14–17)	22 (21–23)	29 (27–31)	23 (21–24)	
>6	4.0 (3.3 - 5.0)	5.6 (4.9 - 6.4)	6.8 (5.4 - 8.1)	6.1 (5.1 - 7.2)	
**Anemia** (Hb < 11 g/dL)	64 (63–66)	70 (69–71)	77 (75–79)	73 (71–74)	<0.001
**Maternal characteristics**					
**Age at first birth** (years)	20.0 (0.07)	19.1 (0.05)	18.7 (0.09)	18.7 (0.07)	<0.001
**Body mass index** (kg/m2)	20.4 (0.09)	19.7 (0.05)	19.5 (0.07)	19.3 (0.09)	<0.001
**Married**	99 (98–100)	99 (98–100)	97 (96–98)	98 (97–99)	0.005
**Smoking** (present)	1.0 (0.4 - 1.6)	1.7 (1.2 - 2.1)	2.5 (1.4 - 3.7)	2.5 (1.8 - 3.2)	<0.001
**Education**					<0.001
No education	32 (29–35)	53 (51–54)	69 (66–72)	58 (56–60)	
Primary	14 (12–15)	14 (13–15)	12 (11–14)	14 (13–16)	
Secondary	44 (42–47)	29 (28–31)	17 (15–20)	25 (24–27)	
**Occupation**					<0.001
Non worker	77 (76–79)	59 (58–61)	33 (30–36)	56 (54–59)	
Manual worker	17 (16–19)	37 (35–38)	64 (61–67)	38 (36–41)	
Non manual worker	5.3 (4.6 - 6.0)	4.0 (3.5 - 4.5)	3.3 (2.5 - 4.1)	5.4 (4.5 - 6.2)	
Higher	10 (9–11)	3.7 (3.2 - 4.2)	1.2 (0.7 - 1.7)	2.2 (1.7 - 2.6)	
**Other characteristics**					
**Residence** (rural)	66 (64–68)	76 (75–78)	91 (89–93)	77 (75–80)	0.035
**Father’s education**					<0.001
No education	20 (18–23)	27 (26–29)	47 (44–50)	32 (30–35)	
Primary	13 (12–14)	15 (14–16)	17 (15–19)	17 (16–19)	
Secondary	49 (47–52)	49 (47–51)	32 (29–35)	44 (42–47)	
Higher	17 (16–19)	8.5 (7.7 - 9.2)	4.2 (3.2 - 5.1)	6.1 (5.2 - 6.9)	
**Household wealth**					
Poor	30 (27–32)	47 (45–49)	77 (75–80)	59 (56–61)	<0.001
Middle	19 (17–20)	23 (21–24)	12 (10–14)	19 (17–20)	
Rich	51 (49–54)	30 (29–32)	10 (8.3 - 12)	23 (21–25)	
**Religion**					<0.001
Hindu	65 (62–69)	84 (82–86)	87 (84–89)	91 (90–93)	
Muslim	30 (26–34)	15 (13–17)	1.3 (0.6 - 1.9)	1.7 (1.0 - 2.4)	
Sikh	1.2 (0.8 - 1.5)	1.0 (0.6 - 1.3)	7.6 (5.8 - 9.4)	1.1 (0.7 - 1.6)	
Christian	2.8 (2.3 - 3.3)	0.4 (0.2 - 0.6)	0.0	2.1 (1.5 - 2.7)	
Other	0.7 (0.4 - 1.0)	0.2 (0.1 - 0.3)	4.5 (3.0 - 6.0)	3.7 (2.6 - 4.7)	

^a^Continuous variables are presented as mean (standard errors) and categorical variables as percentages

The distribution of adult education and household wealth in different levels of anemia is shown in Table 2 (percentages shown are the weighted percentages for survey design). Approximately 69 % (n = 21,786) of the study population was anemic. Of the total anemic population, 26 % (n = 8,772) were mildly anemic, 40 % (n = 12,160) were moderately anemic and 2.9 % (n = 854) were severely anemic. Lowest prevalence of severe anemia (12 %) was in other backward class. Largest anemia population of children was born to un-educated mothers (45 %). Similarly, majority of mild (41 %), moderate (48 %) and severely (55 %) anemic children belonged to un-educated mothers. On contrary, in household with most prevalence of anemia (48 %), father had education up to secondary level. Same was true for mild (49 %), moderate (47 %) and severe (48 %) levels of anemia. The prevalence of overall and severe anemia was highest in poor households (41 % and 45 %, respectively).

**Table 2:** Distribution of caste, adult education and household wealth in levels of anemia^a^

	Anemia levels			
Characteristics	Overall	Mild	Moderate	Severe
N	21786 (69)	8772 (26)	12160 (40)	854 (2.9)
**Caste**				
Other caste	6,024 (28)	2,682 (31)	3,163 (26)	247 (29)
Other backward caste	7,917 (36)	3,098 (35)	4,494 (37)	106 (12)
Scheduled tribe	3,292 (15)	1,329 (15)	1,857 (16)	322 (28)
Scheduled caste	4,556 (21)	1,663 (19)	2,646 (22)	179 (21)
**Maternal education**				
No education	9,906 (45)	3,608 (41)	5,832 (48)	466 (55)
Primary education	3,186 (15)	1,293 (15)	1,768 (15)	125 (15)
Secondary education	7,474 (34)	3,300 (38)	3,941 (32)	233 (27)
Higher education	1,220 (6)	571 (7)	619 (5)	30 (4)
**Father’s education**				
No education	5,835 (27)	2,084 (24)	3,472 (29)	279 (33)
Primary education	3,392 (16)	1,372 (16)	1,899 (16)	121 (14)
Secondary education	10,410 (48)	4,293 (49)	5,704 (47)	413 (48)
Higher education	2,149 (10)	1,023 (12)	1,085 (9)	41 (5)
**Household wealth**				
Poor	9,026 (41)	5,694 (44)	4,367 (36)	383 (45)
Middle	4,486 (21)	2,679 (21)	2,482 (20)	197 (23)
Rich	8,274 (38)	4,641 (35)	5,311 (44)	274 (32)

^a^Analysis has been adjusted for survey specific weights

^a^Estimates are presented as percentages

### Association between caste and anemia

In univariate analysis (model 0), compared to children in other caste, children born in other backward caste, scheduled tribe and scheduled caste were associated with higher prevalence of mild, moderate and severe anemia.

The association of caste with each level of anemia existed after adjusting for childhood (age, sex, birth order, place of residence and religion) and maternal demographic characteristics (BMI, marital status, occupation and smoking status) (model 1).

Further, adjustment for adult education and household wealth, individually (model 2 and model 3, respectively) and simultaneously (model 4) resulted in similar findings (Table 3).

**Table 3:** Association of caste with levels of childhood anemia

		Caste				
		Other caste^a^	Other backward class	Scheduled tribe	Scheduled caste	
Anemia levels		RR (95 % CI)	RR (95 % CI)	RR (95 % CI)	RR (95 % CI)	*p*-trend
**Mild**	Model 0	1	1.08 (1.04 - 1.13)	1.15 (1.10 - 1.22)	1.15 (1.10 - 1.21)	<0.001
	Model 1	1	1.09 (1.04 - 1.13)	1.26 (1.19 - 1.73)	1.17 (1.11 - 1.22)	<0.001
	Model 2	1	1.05 (1.01 - 1.09)	1.18 (1.11 - 1.25)	1.10 (1.05 - 1.16)	<0.001
	Model 3	1	1.08 (1.04 - 1.16)	1.17 (1.10 - 1.24)	1.12 (1.07 - 1.18)	<0.001
	Model 4	1	1.07 (1.02 - 1.11)	1.15 (1.08 - 1.22)	1.10 (1.05 - 1.15)	<0.001
**Moderate**	Model 0	1	1.16 (1.12 - 1.21)	1.25 (1.20 - 1.31)	1.28 (1.23 - 1.34)	<0.001
	Model 1	1	1.15 (1.11 - 1.20)	1.36 (1.29 - 1.43)	1.26 (1.21 - 1.32)	<0.001
	Model 2	1	1.11 (1.07 - 1.15)	1.24 (1.18 - 1.31)	1.19 (1.14 - 1.24)	<0.001
	Model 3	1	1.16 (1.11 - 1.20)	1.25 (1.19 - 1.32)	1.23 (1.18 - 1.29)	<0.001
	Model 4	1	1.13 (1.09 - 1.17)	1.23 (1.16 - 1.29)	1.19 (1.14 - 1.24)	<0.001
**Severe**	Model 0	1	1.46 (1.22 - 1.76)	1.26 (0.99 - 1.60)	2.07 (1.72 - 2.52)	<0.001
	Model 1	1	1.48 (1.23 - 1.79)	1.62 (1.23 - 2.13)	2.09 (1.70 - 2.57)	<0.001
	Model 2	1	1.36 (1.12 - 1.64)	1.38 (1.04 - 1.83)	1.84 (1.49 - 2.27)	<0.001
	Model 3	1	1.46 (1.21 - 1.77)	1.47 (1.11 - 1.96)	2.01 (1.63 - 2.48)	<0.001
	Model 4	1	1.35 (1.12 - 1.63)	1.38 (1.04 - 1.84)	1.87 (1.51 - 2.31)	<0.001

^a^Other caste was considered as reference category

Model 0 Univariate analysis

Model 1 Model 1 + Child age, sex, birth order, religion, maternal age at first child birth, maternal BMI

marital status, residence, occupation and smoking status

Model 2 Model 2 + adult education (maternal and father’s education)

Model 3 Model 2 + household wealth

Model 4 Model 2 + adult education (maternal and father’s education) and household wealth

(Shown are the risk ratio with 95 % confidence interval of developing mild, moderate and severe anemia among other backward castes, scheduled caste and scheduled tribe keeping other caste as reference category)

### Moderating effect of adult education and household wealth

There was no significant interaction between caste and adult education for association with mild, moderate or severe anemia (Maternal education: *p* = 0.41, *p* = 0.35 and *p* = 0.27; Paternal education: *p* = 0.23, *p* = 0.17 and *p* = 0.41 for mild, moderate and severe anemia respectively). Similarly no interaction was observed between caste and household wealth (*p* = 0.97, *p* = 0.39 and *p* = 0.57 for mild, moderate and severe anemia respectively). In stratified analyses on adult education and household wealth, findings remained consistent with overall results (data not shown).

### Sensitivity analyses

First, repeating the analysis for children born to mothers having age ≥ 18 years at the birth of their first child (sample size; n = 24,526 for severe anemia analysis; n = 23,955 for moderate anemia; n = 15,436 for mild anemia) did not change the overall associations (Table 4). Furthermore, we excluded the children of mothers having a BMI < 18.5 kg/m^2^ (sample size; n = 24,369 for severe anemia analysis; n = 23,807 for moderate anemia; n = 15,400 for mild anemia) and re-estimated the association between caste and anemia levels (Table 5). This also did not provide any evidence of a convincing difference.

**Table 4:** Association of caste with levels of childhood anemia for children born to mothers aged ≥ 18 years at first child birth^a^

	Caste				
	Other caste^a^	Other backward class	Scheduled tribe	Scheduled caste	
Anemia levels	RR (95 % CI)	RR (95 % CI)	RR (95 % CI)	RR (95 % CI)	*p*-trend
**Mild**	1	1.05 (1.01 - 1.10)	1.13 (1.05 - 1.23)	1.09 (1.03 - 1.16)	<0.001
**Moderate**	1	1.12 (1.08 - 1.13)	1.22 (1.15 - 1.30)	1.20 (1.13 - 1.25)	<0.001
**Severe**	1	1.34 (1.07 - 1.67)	1.50 (1.05 - 2.14)	2.01 (1.56 - 2.56)	<0.001

^a^Model has been adjusted for child, maternal and household covariates including adult education and household wealth

(Shown are the risk ratio with 95 % confidence interval of developing mild, moderate and severe anemia among other backward castes, scheduled caste and scheduled tribe keeping other caste as reference category)

**Table 5:** Association of caste with levels of childhood anemia for children born to mothers with BMI ≥ 18.5 kg/m2^a^

	Caste				
	Other caste^a^	Other backward class	Scheduled tribe	Scheduled caste	
**Anemia levels**	RR (95 % CI)	RR (95 % CI)	RR (95 % CI)	RR (95 % CI)	*p*-trend
**Mild**	1	1.06 (1.02 - 1.12)	1.14 (1.06 - 1.22)	1.13 (1.07 - 1.20)	<0.001
**Moderate**	1	1.09 (1.04 - 1.14)	1.20 (1.13 - 1.28)	1.20 (1.13 - 1.26)	<0.001
**Severe**	1	1.32 (1.05 - 1.67)	1.34 (1.09 - 1.59)	1.81 (1.39 - 2.34)	<0.001

^a^Model has been adjusted for child, maternal and household covariates including adult education and household wealth

(Shown are the risk ratio with 95 % confidence interval of developing mild, moderate and severe anemia among other backward castes, scheduled caste and scheduled tribe keeping other caste as reference category)

## Discussion

Our results suggest that the caste based social segregation is associated with mild, moderate and severe levels of anemia in India. These associations existed even after accounting for demographic factors including adult education and household wealth. Furthermore, stratification on adult education and household wealth, and using interaction on multiplicative scale for caste, the effect of adult education and household wealth did not differ among different castes.

A number of studies have investigated the caste related health disparities in India. Studies have linked scheduled castes and scheduled tribes with higher comorbidities [13], mortality [15], stunting and wasting in children [19], child mortality [20] and higher prevalence of childhood anemia [21]. Most studies were conducted on regional, small, convenience-based samples except a study by Van de Poel E *et al. *[19] which mainly focused on child stunting and wasting but not anemia. Study by Nayar KR, briefly reported the prevalence of childhood anemia using nationally representative NFHS 1998–1999 data (ref.). In present study, using more recent data (NFHS 2005–2006), we examine the association between caste and childhood anemia independent of adult education and household wealth. In addition, we investigate effect modifying role of parental education and wealth in the association between caste and childhood anemia. To our knowledge, this is the first study to investigate the association of caste with childhood anemia independent of adult education and household wealth in a nationally representative sample of children younger than 5 years in India.

Health disparities exist across various other population subgroups also in developed countries. For instance, low birth weight and infant mortality is more strongly associated with African-American race than other racial/ethnic groups in the United States (US) [22]. Similar to disadvantageous castes in India, racial/ethnic minorities (except Asian-Americans) in the US usually belong to low socioeconomic groups and face discrimination. Studies suggest socioeconomic status, racial discrimination and genetic factors as among the potential causes of poor health in African-Americans [23]. However, unlike race/ethnicity, there is no reported genetic difference in castes in India and therefore is unlikely to be a cause of disproportionate childhood anemia in disadvantageous caste in India.

Most of people of disadvantageous cates belong to low socioeconomic groups in India. Low socioeconomic status may affect the prevalence of anemia *via* several pathways including 1) poor living and working conditions, 2) adverse health behaviors such as maternal smoking poor dietary habits and 3) limited health car use and limited health literacy which might influence their noncompliance with use of iron supplements. In our data also, more women were smokers in disadvantageous castes than in other caste. Thereby, these pathways might explain the association between caste and childhood anemia. However, in our study, after adjusting for socioeconomic factors (*i.e.* adult education and household wealth) the association between caste and childhood anemia was still significant. It suggests that independent of socioeconomic status there are other pathways that link caste to childhood anemia. We believe that caste based discrimination might be one such pathway that associate caste with childhood anemia. It should be noted that NFHS 3 do not have data on caste based discrimination and investigating role of caste based discrimination in the association between caste and childhood anemia is beyond the aims of this study. Future studies should investigate this issue.

After independence in 1947, affirmative government actions promoted reservations in government educational institutes and jobs for disadvantageous caste to ensure better education and job opportunity. Such opportunities improved the level of educational attainment and economic gains in disadvantageous caste [24]. The adult education and household wealth are known determinants of health. Adult education and household wealth influence health in a number of ways including access to better health care, improved health literacy and life style habits. Therefore, adult education and household wealth can be considered as important effect modifiers in association between caste and health conditions like childhood anemia. However, in our results, caste reservation for ensuring better education and economic opportunities could not completely mitigate the inequalities in occurrence of childhood anemia between different castes. In order to explore the effect modifying role of adult education and household wealth, we also stratified analysis by these factors and did not observe differential association of caste for different levels of adult education and household wealth. One of the important finding in this study was that there was no consistent linear relationship between caste and childhood anemia. This might suggest that the amount of social disadvantage do not increase linearly in other backward class, scheduled tribe and scheduled caste in relation to other castes.

Strengths of this study are the use of detailed information on a number of relevant covariates from large nationally representative data. Our study has certain limitations. Because of data constrains we had limited information on some of the relevant covariates. There was unavailability of information on maternal psychological factors that are known determinant of childhood anemia. We also lacked information on access to healthcare which is linked with malnutrition in childhood [19]. Since in Indian settings, place of residence (rural/urban) is used as a proxy of access to health care, we accounted for place of residence. Therefore, limited information on health care access is less likely to affect our results. Caste reservation policy in India started well before the collection of data on demographic and health indicators of the population. Therefore we were not able to compare the gains (if any), resulting from such policies in mitigating caste based inequalities in prevalence of childhood anemia. Eventually it is difficult to conclude whether caste reservation has helped in countering health related disadvantage in lower caste.

This study has important public health implications. As our results suggested that there is a higher risk of anemia in disadvantageous caste and these caste based disparities in childhood anemia exist independent of adult education and household wealth public health approaches tailored to disadvantageous castes irrespective of their educational and wealth status might be more beneficial in countering childhood anemia in India. Eleventh five year plan for anemia control for children in India can also be benefited from targeted IFA (Iron-Folic Acid) supplements to disadvantageous caste. Furthermore, future studies should investigate causal pathways that link caste to childhood anemia.

## Conclusion

Caste is an independent determinant of childhood anemia in India even after accounting for other social advantages including adult education and household wealth. Future public health actions targeted to disadvantaged castes might be more beneficial in countering childhood anemia in India.
